# Supplementary material for: Role of CCK1 receptor in metabolic benefits of intestinal enteropeptidase inhibition in mice
Source: PLoS One. 2025 Jun 3;20(6):e0312927. doi: 10.1371/journal.pone.0312927 (PMC12132930; doi:10.1371/journal.pone.0312927)
Supplement: S1 Table — Data represents the mean SEM of measurements from 9–10 animals for each group. No statistical differences were observed comparing by genotype or treatment (One Way ANOVA+Tukey test). N.B. Glycine and citrulline were also quantified for each sample, however, were not detectable in all samples & thus not reported below. (PDF) [file pone.0312927.s001.pdf]

Supplementary Table 1. Plasma Amino Acids (μM) were measured in terminal fed blood samples from HFD-fed WT and CCK1R KO mice treated for 28 days with camostat metabolite (FOY-251) admixture or pair-fed (PF) to the same amount. Data represents the mean SEM of measurements from 9-10 animals for each group. No statistical differences were observed comparing by genotype or treatment (One Way ANOVA+Tukey test). N.B. Glycine and citrulline were also quantified for each sample, however, were not detectable in all samples & thus not reported below.

	**WT-Control**	**WT-FOY-251**	**CCK1R KO Control**	**CCK1R KO-FOY-251**	**WT-Pair Fed**	**CCK1R KO-PF**
Ala	0.567±0.051	0.643±0.041	0.672±0.045	0.771±0.062	0.738±0.076	0.675±0.061
Ser	0.028±0.003	0.033±0.004	0.030±0.004	0.035±0.003	0.041±0.005	0.037±0.004
Pro	13.0±1.0	14.0±1.0	16.1±1.6	15.2±1.0	18.2±2.0	17.1±2.2
Pro, 4OH-	0.050±0.006	0.055±0.005	0.044±0.004	0.059±0.010	0.058±0.006	0.054±0.005
Val	17.1±1.0	17.6±0.8	18.7±1.2	16.9±0.6	18.2±1.4	17.4±1.5
Thr	0.481±0.046	0.384±0.036	0.417±0.031	0.318±0.029	0.398±0.048	0.313±0.032
Orn	0.080±0.009	0.093±0.009	0.098±0.008	0.083±0.009	0.114±0.012	0.094±0.009
Gln	0.426±0.025	0.488±0.033	0.497±0.031	0.517±0.035	0.508±0.026	0.477±0.021
Lys	0.343±0.044	0.401±0.053	0.324±0.033	0.372±0.043	0.449±0.057	0.334±0.049
Met	17.0±0.8	20.0±1.2	21.3±1.6	23.5±1.1	23.9±2.0	21.4±1.7
His	5.30±0.33	5.52±0.23	6.21±0.30	6.80±0.42	6.44±0.33	5.98±0.22
Phe	25.0±1.5	25.1±1.6	29.0±4.0	26.4±2.0	25.2±2.1	23.7±2.3
Arg	0.228±0.051	0.354±0.039	0.407±0.101	0.642±0.069	0.397±0.091	0.423±0.078
Tyr	21.8±1.5	19.1±1.6	19.0±1.0	18.8±1.5	25.2±1.6	21.8±1.5
Trp	20.7±1.9	20.2±1.5	20.5±2.3	16.8±1.1	26.2±2.7	21.9±1.5
5-HT	1.54±0.44	1.56±0.39	1.50±0.57	1.48±0.83	0.88±0.43	0.79±0.38
Ile/Leu	26.7±1.4	28.9±1.9	39.2±4.8	32.1±2.5	31.8±2.4	31.4±3.7
