# Supplementary material for: Role of CCK1 receptor in metabolic benefits of intestinal enteropeptidase inhibition in mice
Source: PLoS One. 2025 Jun 3;20(6):e0312927. doi: 10.1371/journal.pone.0312927 (PMC12132930; doi:10.1371/journal.pone.0312927)
Supplement: S2 Table — Data represents the mean SEM of measurements from 9–10 animals for each group. No statistical differences were observed comparing by genotype or treatment (One Way ANOVA+Tukey test). Plasma liver enzymes (ALT = alanine transaminase, AST = aspartate transaminase), BHB (β-hydroxybutyrate), Cholesterol, HDL-C, NEFA (non-esterified fatty acids) and triglycerides were measured using an Alfa Wassermann blood chemistry analyser. (PDF) [file pone.0312927.s002.pdf]

S2 Table. Clinical chemistry of terminal fed blood samples from HFD-fed WT and CCK1R KO mice treated for 28 days with camostat metabolite (FOY-251) admixture or pair-fed (PF) to the same amount. Data represents the mean SEM of measurements from 9-10 animals for each group. No statistical differences were observed comparing by genotype or treatment (One Way ANOVA+Tukey test). Plasma liver enzymes (ALT= alanine transaminase, AST= aspartate transaminase), BHB (β-hydroxybutyrate), Cholesterol, HDL-C, NEFA (non-esterified fatty acids) and triglycerides were measured using an Alfa Wassermann blood chemistry analyser.

	**ALT U/L**	**AST U/L**	**BHB mmol/L**	**Cholesterol mg/dL**	**HDL mg/dL**	**NEFA mg/dL**	**Triglyceride mg/dL**
WT-Control	248.3±62.5	546.5±140.6	0.40±0.03	194.9±8.5	102.0±9.8	1.04±0.49	170.9±14.7
WT-FOY-251	159.1±17.7	368.3±68.8	0.33±0.03	171.4±7.0	99.8±5.4	0.33±0.02	204.0±20.2
CCK1R KO-Control	178.0±39.7	731.6±200.9	0.76±0.22	200.3±7.1	75.3±15.6	2.6±0.64	214.2±24.6
CCK1R KO-FOY-251	102.5±32.5	358.0±80.3	0.57±0.14	178.5±5.7	75.1±13.5	1.33±0.28	215.2±33.9
WT-PF	245.7±57.4	421.8±92.8	0.38±0.06	200.6±6.1	187.3±101.5	0.78±0.41	161.4±20.9
CCK1R KO-PF	173.3±44.3	379.0±60.6	0.36±0.06	179.1±5.9	94.6±11.3	1.33±0.47	149.4±22.7
